# NK cell-based cancer immunotherapy: from basic biology to clinical development

**DOI:** 10.1186/s13045-020-01014-w

**Published:** 2021-01-06

**Authors:** Sizhe Liu, Vasiliy Galat, Yekaterina Galat4, Yoo Kyung Annie Lee, Derek Wainwright, Jennifer Wu

**Affiliations:** 1 Department of Urology, Feinberg School of Medicine, Northwestern University, 303 E. Superior St., Lurie Research Building 6-117, Chicago, IL 60611 USA; 2 Department of Pathology, Feinberg School of Medicine, Northwestern University, Chicago, IL USA; 3 Robert H. Lurie Comprehensive Cancer Center, Northwestern University Feinberg School of Medicine, Chicago, IL USA; 4 Department of Pediatrics, Stanley Manne Children’s Research Institute, Ann & Robert H. Lurie Children’s Hospital, Northwestern University Feinberg School of Medicine, Chicago, IL USA; 5 Biology Program, Emory University, Atlanta, USA; 6 Departments of Neurological Surgery, Medicine-Hematology and Oncology, Microbiology-Immunology, Feinberg School of Medicine, Northwestern University, Chicago, IL 60611 USA; 7 Institute of Theoretical and Experimental Biophysics, Russian Academy of Sciences, Pushchino, Russia

**Keywords:** NK cell, Cancer immunotherapy, Clinical trials, iPSC

## Abstract

Natural killer (NK) cell is a specialized immune effector cell type that plays a critical role in immune activation against abnormal cells. Different from events required for T cell activation, NK cell activation is governed by the interaction of NK receptors with target cells, independent of antigen processing and presentation. Due to relatively unsophisticated cues for activation, NK cell has gained significant attention in the field of cancer immunotherapy. Many efforts are emerging for developing and engineering NK cell-based cancer immunotherapy. In this review, we provide our current understandings of NK cell biology, ongoing pre-clinical and clinical development of NK cell-based therapies and discuss the progress, challenges, and future perspectives.

## Background

Natural killer (NK) cells are an essential part of tumor immunosurveillance, evidenced by higher cancer susceptibility and metastasis in association with diminished NK activity in mouse models and clinical studies [1–3]. Using an array of germline-encoded surface receptors, NK cells are able to recognize and rapidly act against malignant cells without prior sensitization. Upon activation, NK cells release cytotoxic granules containing perforin and granzymes to directly lyse tumor cells, in a similar fashion to activated cytotoxic T cells. NK cells are also potent producers of chemokines and cytokines such as interferon gamma (IFN-γ) and tumor necrosis factor alpha (TNF-α) and thereby are essential in modulating adaptive immune responses. Due to their innate ability to eliminate tumor cells, NK cell-based immunotherapies against cancer have been investigated for decades. Early clinical trials have demonstrated the overall safety of NK cell infusion, even in the allogeneic setting [4–7]. The feasibility of utilizing allogeneic NK cells, the established safety profiles, and the fast-acting nature of NK cells largely have led to the emerging effort to develop “off-the-shelf” NK cell-based cancer immunotherapy. However, there are many challenges to overcome, such as difficulty to meet clinical-grade ex vivo expansion, limited in vivo persistence, limited infiltration to solid tumors, and tumor editing to evade NK cell activity. Various strategies are being employed to overcome these challenges to improve the efficacy of NK cell-based therapy, such as ex vivo pre-conditioning with cytokines and/or small molecular drugs, engineering an “off-the-shelf” or iPSC-differentiated chimeric antigen receptor (CAR)-NK. There has been an explosion of NK-based immunotherapies in pre-clinical development and clinical development. Herein, we will provide an updated overview of the emerging endeavors for developing NK cell-based cancer immunotherapy from pre-clinical conceptual development, clinical grade expansion, and ongoing clinical development.

## Main text

### NK cell biology

NK cells were identified over four decades ago as lymphocytes with innate ability to lyse tumor cells without the need for prior sensitization [8–10]. NK cells can trigger target cell death by releasing cytotoxic granules containing granzymes and perforin and through death receptor-mediated pathways (e.g., FasL/Fas) [11]. NK cells also play immunomodulatory functions by secreting chemokines and cytokines, such as RANTES and IFN-γ [12, 13].

In humans, NK cells are traditionally identified by the absence of CD3 and the presence of CD56 on their surface as characterized by flow cytometry. In mouse, the lack of CD3 and the presence of NK1.1 are canonical criterion for distinguishing NK cells. In mouse strains lacking NK1.1 expression (e.g., BALB/c), CD49b is used for NK cell identification. The natural cytotoxic receptor NKp46 is also often used to identify mouse and human NK cells in combination with the absence of CD3 expression. Notably, certain tissues such as the mucosal barriers possess subsets of recently identified innate lymphoid cells (ILCs) that also share canonical markers of NK cells. For example, a subset of IL-22 secreting human ILC3s is CD56^+^NKp46^+^CD3^−^ [14]. Additional markers such as lack of c-kit can be used to distinguish human NK (c-kit-) from ILC3s (c-kit +) [14]. In mouse, NK and ILC1 are NK1.1^+^CD3^−^ but can be further characterized by CD49a and Eomes expression. NK cells are CD49a^−^Eomes^+^, whereas ILC1s are CD49a^+^Eomes^−^ [15, 16].

NK cells are found both in blood at levels of 5%-15% of circulating lymphocytes and in various lymphoid and non-lymphoid organs such as the spleen, lung, and liver [17, 18]. Based on characterization of NK cells in the peripheral blood, human NK cells are conventionally sub-divided into two major subsets: CD56^bright^CD16^dim/−^ and CD56^dim^CD16^+^, with the former classically believed to be less mature and a potent cytokine producer and the latter more mature and the most cytotoxic [17, 19]. Most NK cells in the blood are CD56^dim^, whereas the CD56^bright^ subset only represents less than 15% of total circulating NK cells [17]. The relative proportion of CD56^bright^ and CD56^dim^ NK cells in tissues can be very different from that observed in the peripheral blood [17]. Notably, many tissue-resident NK subsets are now shown to be phenotypically and functionally distinct from conventional peripheral blood NK cells [20]. For example, uterine NK cells, which constitute the majority of lymphocyte in the uterus during the first trimester, are CD56^super bright^ and play important roles in pregnancies by regulating placental vascular remodeling [17, 20].

Conventional NK cells are short-lived innate lymphocytes that lack antigen specificity. Recent studies revealed that subset of mature NK cells can elicit long-lived “adaptive”-like nature in the specific context of CMV infection [21]. It is now understood that the “adaptive”-like NK cells express the activating receptor NKG2C and that the “adaptive” nature of NKG2C^+^ NK cells attributes to the non-classical MHC I molecule HLA-E presenting CMV-specific viral peptide to NKG2C [22]. The NKG2C^+^NK cells can be found in the circulation of HCMV-seropositive individuals [22].

NK cells originate from CD34^+^ hematopoietic stem cells. Bone marrow is considered to be the primary site of NK development. More recent evidence had indicated that NK cells can also develop and mature at secondary lymphoid organs, including tonsils, spleen, and lymph nodes [19]. NK cell progenitors progress through distinct developmental stages and gradually acquire the expression of surface receptors that define NK cell identity such as NK1.1 and CD56 and/or regulate their effector functions such as CD16 and NKp46 [19]. Distinct from T cells, there is no master transcriptional factor that controls NK cell development. Instead, a combination of transcription factors, including T-bet, Eomes, E4BP4, Id2, and BLIMP, was identified to instruct NK development and maturation [23]. Common gamma chain cytokines such as IL2, IL-7, and IL15 and their receptor components including CD122 and CD127 have been shown to play essential roles in NK development and homeostasis [19]. Despite decades of work, the ontogeny of NK cells in humans is still not fully elucidated. The conventional linear model suggests that mature NK cells arise from common lymphoid progenitors (CLPs) by progressing through a linear continuum [24]. The linear model proposes that CD56 marks a transition from immature into a more mature status and that immature CD56^bright^ NK cells further differentiate into mature CD56^dim^ populations in human [19, 24]. Recent evidence has challenged this model and suggests a possibility of more branched development in the form of both CLPs and common myeloid progenitors (CMPs) giving rise to NK cell progenitors. The branched model also proposes that distinct precursor populations independently develop into different mature NK subsets [24].

### NK cell receptors and NK activation

NK cells are mounted with a repertoire of inhibitory and activating surface receptors (Table 1) [25–29]. Distinctly different from T cell receptors, NK cell receptors are germline-encoded and “hard-wired” receptors without a requirement for “V(D)J” recombination. Upon ligation, these receptors transmit either inhibitory or activating signals to control NK activation. The integration and balance of the activating and inhibitory signals from the ligand/receptor interactions dictates the status of NK cell activation. For instance, healthy cells express no or minimal level of ligands for NK cell activating receptors, but express high levels of the major histocompatibility complex class I molecules (MHC I), also known as human leukocyte antigen (HLA), that ligates to the killer immunoglobulin-like (KIR) family inhibitory receptors on NK cells to protect them from NK attack [29]. Conversely, tumorigenic cells or virally infected cells have downregulated MHC I expression but upregulated levels of ligands for NK cell activating receptors and thus trigger NK cell activation due to the lack of inhibitory signals and/or the presence of activating signals [11]. In allogeneic transfer settings, the concept of KIR and HLA mismatches between donor and recipient was the original strategy aimed at enhancing the activation of donor NK cells to eradicate patient’s tumor cells. NK cells also play a significant role in antibody-mediated cancer therapies by utilizing the Fcγ receptor to carry out antibody-dependent cellular cytotoxicity (ADCC) [30].

**Table 1:** NK cell receptors and their ligands in human

	Ligands
*NK activating receptor*	
NKG2D	MHC class I chain-related protein A (MICA) and B (MICB), UL16-binding proteins (ULBP1-6)
DNAM1	PVR(CD155), nectin-2 (CD112)
NKp30 (NCR3)	pp65, B7-H6, galectin-3, BAG6, viral hemagglutinin (HA), heparan sulfate (HS) glycosaminoglycans (GAGs), (DBL)-1a domain of *Plasmodium falciparum* erythrocyte membrane protein-1
NKp44 (NCR2)	PDGF-DD, 21spe-MLL5, PCNA, Syndecan-4, Nidogen-1, viral HA, HS GAGs
NKp46 (NCR1)	Complement factor P, viral HA, HS GAGs, (DBL)-1a domain of *Plasmodium falciparum* erythrocyte membrane protein-1, vimentin
CD16 (FcγRIII)	Fc portion of IgG antibodies
*NK inhibitory receptors*	
CD94/NKG2A	HLA-E
KIR2DL1	HLA-C, group 2
KIR2DL2/3	HLA-C, group 1
KIR3DL1	HLA-Bw4
KIR3DL2	HLA-A3, A11

### Preclinical development of NK cell-based cancer immunotherapy

Current preclinical development of NK cell-based therapy was largely inspired by early clinical studies. With the understanding of how NK cells are activated, the initial NK cell-based therapy was pioneered in the clinical setting of hematopoietic stem cell transplants (HSCTs) whereby NK cells were shown to have the capacity to exert a graft versus leukemia effect. The Ruggeri group showed that KIR-mismatched alloreactive donor NK cells protected bone marrow-transplanted AML patients from AML relapse while sparing graft versus host diseases (GVHD) [31, 32]. Miller et al. further pioneered the use of NK cells in non-transplant settings. They showed that infusion of NK cells from HLA-haploidentical donors combined with subcutaneous IL-2 administration after a pre-conditioning regimen of high-dose cyclophosphamide and fludarabine resulted in successful in vivo expansion of donor NK cells and the induction of complete remission in 5 out of 19 patients with poor-prognosis acute myeloid leukemia (AML) [5]. Miller et al. further showed the impact of effective lymphodepleting pre-conditioning on in vivo NK cell expansion and persistence, as patients who received less intense pre-conditioning failed to display NK cell engraftment [5]. It is currently believed that the success of adoptive transfer requires the creation of a lymphopenic environment to provide a niche for donor cells to survive and proliferate.

Initial successes of adoptive NK cell transfer in treating hematological cancers prompted clinical endeavors in using the strategy against solid cancers. NK cells are cytotoxic against a wide range of tumor cells of solid cancer types in vitro. Anti-tumor activities of adoptively transferred NK cells in vivo have been demonstrated as well in pre-clinical xenograft mouse models of ovarian cancer, glioblastoma, and metastatic colorectal cancer [33–36]. The safety of NK cell-based therapy has been demonstrated in both autologous and allogeneic haploidentical settings [4–7]. Clinical efficacy of this strategy has proven to be thus far be limited. The following section summarizes the current pre-clinical efforts to enhance the efficacy of NK cell-based therapy.

### CAR-NK cell as an alternative to CAR-T therapy

T cells equipped with CARs have been shown to provide clinical benefit for patients with select liquid cancers. Two CD19-targeting CAR-T products were approved by the FDA for treatment of B cell lymphomas and acute lymphoblastic leukemia (ALL). However, CAR-T therapy has two major challenges: (i) requirement of a substantiate length of time to generate a therapeutic dose of autologous CAR-T cells which limits its feasibility to treat patients with rapidly progressing diseases; (ii) difficulty to obtain sufficient number of autologous T cells for CAR-T cell generation from heavily pre-treated and often lymphopenic cancer patients [37]. As an alternative to CAR-T cell therapy, CAR-NK cell therapy not only circumvents these challenges but also presents additional major advantages: (i) the ability to use unlimited allogeneic NK source without concern of GVHD [6, 31, 32]; (ii) the potential to generate “off-shelf” product with NK cell line or iPSC-NK [37–41]; (iii) relatively shortened production time; iv) recognition and killing tumor cells through NK cell native receptors independent of the CAR engineering [42], less likely allowing disease escape through downregulation of the CAR antigens as shown with CAR-T cell therapy [37].

Similar to CAR-T cells, CAR-NK cells are genetically modified to express CARs that recognize a specific antigen uniquely expressed or overexpressed by target cells. In most pre-clinical studies, lentiviral or retroviral-based transduction was used to achieve stable and sustained CAR expression in NK cells. Non-viral vector-based delivery methods such as transposon systems and electroporation of mRNA have been used as well [41, 43–45]. A wide range of tumor antigens have been targeted by CAR-NK cells in pre-clinical studies for hematological malignancies and solid tumors [38, 39, 41–76]. They are also summarized in Table 2. The antigen recognition domain usually consists of a single-chain fragment (scFV) molecule derived from a monoclonal antibody, and nanobody-based constructs have been used in limited studies to date [72]. For hematological cancers, CD19 remains a major target. Antigens such EGFRvIII, mesothelin, and Her2 have been targeted by CAR-NK cells for the treatment of solid cancers including colorectal cancer, ovarian cancer, breast cancer, and glioblastoma [41, 48]. The signaling domains of CAR-NK cells are very similar to those in CAR-T cells, typically composed of the fusion of CD3ζ with one or two TCR co-stimulatory molecule (s), such as CD28, 4-1BB, 2B4, DNAM1, and NKG2D. Among these TCR cell co-stimulatory molecules, 4-1BB, DNAM1, 2B4, and NKG2D were also expressed by NK cells as native activating receptors. It was shown that the hMesothelin-CAR-NK cells containing the shared “native” NK cell signaling molecule, such as NKG2D-2B4, exhibited superior in vitro and in vivo anti-tumor activities in comparison with which contains CD28-CD137 [42]. The NKG2D-2B4 containing CAR-NK had elevated Syk and Erk1/2 phosphorylation [42].

**Table 2:** CAR-NK cells that have been evaluated preclinically

Target	CAR construct(s)	Source of NK	Method(s)	Cancer type(s)	References
*Hematological cancers*					
CD19	CD19-scFv-CD3ζ, CD19-scFv-CD28-CD3ζ, CD19-scFv-41BB-CD3ζ, CD19-scFv-DAP10-CD3ζ, (iC9*).CD19-scFv.CD28-CD3ζ-(IL15*)	NK-92, NKL, Cord Blood, peripheral blood	Retrovirus, lentivirus	B cell malignancies	[38, 39, 49, 52, 63]
FLT3	FLT3-scFV-CD28-CD3ζ	NK-92	Lentivirus	B cell acute lymphoblastic leukemia (B-ALL)	[62]
CS1	CS1-scFv-CD28-CD3ζ	NK-92	Lentivirus	Multiple Myeloma	[53]
CD38	Nb(CD38)^a^-CD28-41BB-CD3ζ	NK-92 (with CD38 knocked out)	Retrovirus	Multiple Myeloma	[72]
CD4	CD4-scFv-CD28-41BB-CD3ζ	NK-92	Lentivirus	Peripheral T cell lymphoma	[73]
CD5	CD5-scFv-2B4-CD3ζ	NK-92	Lentivirus	T cell malignancies	[46]
CD7	Nb(CD7)-CD28-41BB-CD3ζ	NK-92MI	PiggyBac Transposon System	T cell acute lymphoblastic leukemia (T-ALL)	[67]
*Solid cancers*					
Wild Type EGFR and/or EGFRvIII	EGFR-scFv-CD28-CD3ζ, EGFR-scFv-CD28-41BB-CD3ζ, EGFRvIII-scFv-CD28-CD3ζ, EGFRVIII-scFv-DAP12	NK-92 NKL, YTS	Lentivirus	Glioblastoma, renal cell carcinoma, breast cancer	[47, 51, 58, 59, 71, 76]
ErbB2/HER2	ErbB2-scFv-CD3ζ, ErbB2-scFv-CD28-CD3ζ, ErbB2-scFv-41BB- CD3ζ	NK-92	Lentivirus, Retrovirus	Breast carcinoma, glioblastoma	[48, 60, 70]
GD2	GD2-scFv-CD3ζ	NK-92	Retrovirus	Neuroblastoma, melanoma, breast carcinoma	[65]
Glypican-3 (GPC3)	GPC3-scFv-CD28-41BB-CD3ζ, GPC3-scFv-CD3ζ, GPC3-scFv-CD28-CD3ζ, GPC3-scFv-DNAM1-CD3ζ, GPC3-scFv-DNAM1-2B4-CD3ζ	iPSC, NK-92	Lentivirus	Ovarian cancer, hepatocellular cancer	[57, 66]
EpCAM	EpCAM-scFV-41BB-CD3ζ, EpCAM-scFv-CD28-CD3ζ	NK-92	Lentivirus	Colorectal Cancer	[47, 50, 61]
Mesothelin	Mesothelin-scFv-(NKG2D*)^b^-2B4-CD3z, Mesothelin-scFv-CD28-41BB-CD3ζ	NK-92, iPSC	PiggyBac Transposon System; Lentivirus	Ovarian cancer	[42, 75]
Prostate Stem Cell Ag (PSCA)	PSCA-scFv-DAP12	YST cell line, primary NK	Lentivirus	Prostate Cancer	[55]
Carcinoembryonic antigen (CEA)	CEA-scFv-CD3ζ	NK-92MI	Retrovirus	Colorectal Cancer	[69]
CD133	CD133-CD28-41BB-CD3ζ	NK-92	Lentivirus	CD133 + cancer stem cells, ovarian cancer	[64]
c-MET	c-MeT-scFv-41BB–DAP12	Peripheral blood	Lentivirus	Liver cancer	[56]
NKG2D ligands	NKG2D-CD3ζ, NKG2D-CD28- CD3ζ, NKG2D-CD28-41BB-CD3ζ, NKG2D-DAP10-CD3ζ	Peripheral blood, NK-92	RNA electroporation, PiggyBac Transposon System	NKG2DL + cancer cells, Ovarian cancer	[43, 44, 54]
PD-L1	PD1-(NKG2D*)^c^-41BB	NK-92	Lentivirus	PD-L1 + tumor cells	[68]
Tissue Factor (TF)	Factor VII light chain (FvIIL)-CD28-41BB-CD3ζ	NK-92MI (transduced to express CD16)	Lentivirus	Triple-negative breast cancer	[74]

For CAR constructs, only the antigen recognition domain and signaling domain(s) are listed unless otherwise specified. Other functional domains will be in parentheses and marked by asterisks (*). Abbreviations: scFv = single-chain variable fragment; FLT3 = FMS-like tyrosine kinase 3;

^a^Nanobody for CD38

^b^transmembrane domain of NKG2D

^c^hinge region and transmembrane domain of NKG2D

Beyond directing CAR-NK cytotoxicity against tumor cells by targeting tumor antigens, it has been proposed that CAR-NK cells can be used to eliminate immunosuppressive immune cells in the tumor microenvironment that include myeloid-derived suppressor cells (MDSCs) and M2 tumor-associated macrophages (TAMs) [77, 78]. Parihar and colleagues designed CAR-NK cells expressing the activating receptor NKG2D as the antigen recognition to target human MDSCs. They demonstrated in vitro and in a mouse xenograft model grafted with human neuroblastoma and MDSCs that NKG2D CAR-NK cells can reduce human MDSCs efficiently [77]. Moreover, the CAR-NK cells secrete pro-inflammatory cytokines and chemokines which may improve the infiltration and functions of subsequently infused CAR-T cells in the mouse model [77]. It has been proposed that CAR-NK cells may be able to be combined with T cell-based therapies for solid tumors [77].

### Killer cell engagers unleash NK cytotoxicity against tumor cells

Although CAR-NK cells hold great promise as a future “off-the-shelf” drug, there are NK cell-specific challenges, such as potential loss of viability and/or activity with freeze–thaw process and lack of in vivo persistence. To circumvent these challenges, Bi- and tri-specific killer engagers, BiKEs and TriKEs, are in development as a complemental approach. The BiKEs or TrikEs are composed of two or three linked single-chain antibody variable fragments of different antigen specificities [79]. The current in-development BiKEs or TrikEs simultaneously engage with CD16 and tumor antigens, thereby inducing the formation of immune synapses and NK-mediated ADCC [79]. In pre-clinical studies, various BiKEs and TriKEs have been designed to target a number of tumor antigens: CD19, CD20, and CD33 for hematological cancers, HER2, EGFR, and EpCAM for solid tumors [79]. Cytokines, such as IL15, have been incorporated into killer engagers to further enhance NK cell functions [80]. Vallera et al. reported that a CD16/IL-15/CD33 TriKE not only enhances NK-mediated immunity against CD33^+^ targets, but also promotes the in vivo persistence, activation, and survival of NK cells by delivering IL-15 [80]. This TriKE design (GTB-3550) is currently in clinical trial for treating high-risk hematological malignancies (NCT03214666). Tri-functional NK cell engagers (NKCEs), which crosslink both NKp46 and CD16, have demonstrated superior in vitro and in vivo anti-tumor activities compared to conventional monoclonal antibodies targeting the same antigens [81].

### NK cells “priming” strategies

While the CAR technology seeks to enhance NK cell functions by genetically directing their target specificity, there are active investigations on other strategies to effectively “prime” NK cells ex vivo or in vivo for optimal anti-tumor functions after their infusion. It has been shown that freshly isolated, resting NK cells are generally less lytic as compared to NK cells primed via various strategies [82].

Cytokine-mediated activation is frequently employed and currently under extensive investigation. In many studies, IL-2 or IL-15 is supplemented during ex vivo expansion of NK cells and sometimes after NK cell infusion as well [5]. ALT-803, an IL-15/IL-15Ra fusion complex, was shown to enhance NK cell functions both in vitro and in vivo [83]. Short-term (18–20 h) pre-activation with ALT-803 augmented cytotoxicity and ADCC of NK cells in vitro. In a xenograft mouse model of lymphoma, co-administration of ALT-803 with NK cells significantly enhanced anti-CD20-triggered, NK cell-mediated ADCC effects [83]. Recently, several groups demonstrated that mouse and human NK cells pre-activated with a cocktail of IL-12/15/18 had enhanced and sustained anti-tumor effector functions in vitro and in vivo after infusion [84–86]. It was proposed that the cytokine pre-activated NK cells were “memory-like” with an enhanced response to cytokine or activating receptor re-stimulation weeks or months after the initial pre-activation [84]

In addition to cytokine-only strategies, Cichocki and colleagues demonstrated that pharmacologic inhibition of GSK3 kinase with CHIR99021 drives late-stage maturation of ex vivo-expanded human peripheral blood NK cells and enhanced their anti-tumor effector functions both in vitro and in vivo [87]. Human NK cells expanded with IL-15 in the presence GSK3 inhibition had increased expression of the NK cell maturation marker CD57 and transcription factors associated with late-stage NK cell maturation including T-bet, ZEB2, and BLIMP-1 as compared to those expanded with IL-15 alone [87]. The expanded human NK cells showed potent ADCC activities in vitro and superior tumor control in a mouse xenograft model of ovarian cancer when combined with Herceptin, an anti-HER2 antibody [87]. This strategy of GSK3 inhibition was used to generate FATE-NK100, which is being evaluated in an ongoing phase 1 clinical trial either as a monotherapy or in combination with monoclonal antibodies in patients with advanced solid tumors (NCT03319459).

While the majority of investigation of ex vivo NK priming strategies rely on one or more cytokines, some studies indicate that cytokine supplement is not as critical for NK cell priming. One study showed that overnight co-incubation with a leukemia cell line CTV-1 or its lysate without cytokines was sufficient to prime potent NK cells in vitro cytotoxicity against otherwise NK-insensitive tumor cells but not normal hemopoietic cells [88]. The mechanism of CTV-1-mediated NK priming is unclear. It was suggested that induced expression of CD69 on NK cells is important for the cytotoxicity of the CTV-1 tumor cell-activated NK [88]. Based on the pre-clinical findings, CTV-1 lysate-primed human NK cells (CNDO-109-NK cells) derived from HLA-haploidentical donors were evaluated for safety in a phase I clinical trial (NCT01520558) in high-risk AML patients with AML after first complete remission [89]. CNDO-109-NK cells were found well tolerated without occurrence of dose-limiting toxicities [89]. Three out of 12 patients had durable complete remissions [89], providing evidence for further clinical evaluation of this approach.

### Methods to enhance the infiltration and functions of infused NK cells

Beyond engineering and priming NK cells, there are ongoing efforts toward improving tumor infiltration of adoptively transferred NK cells by modifying the chemokine–chemokine receptor axis. Lee et al*.* developed an NK cell recruiting protein-conjugated antibody (NRPbody) containing a mesothelin-specific tumor targeting domain Meso-scFv and the chemokine CXCL16 linked by a furin cleavage sites [90]. Mesothelin is a tumor differentiation antigen that is highly overexpressed in several human cancers including malignant mesothelioma, pancreatic, ovarian, and lung adenocarcinoma [91]. It was hypothesized that once bound to mesothelin-overexpressing tumor cells, furin-mediated cleavage would release CXCL16 from the NRPbody and thereby recruit NK cells to the tumor sites [90]. The cleavable CXCL16 containing NRPbody was shown to promote NK cell migration in vitro and infiltration into the tumor sites in vivo in xenografted mouse models of orthotopic and metastatic pancreatic cancer [90]. In the xenograft models, NK cell infusion combined with intraperitoneal injection of the NRPbody significantly reduced tumor burden as compared to NK infusion combined with the non-cleavable control [90]. It remains to be determined how NRPbody will interact with a more complex and realistic immune contexture in the TME which consists of more than NK cells, as CXCL16 was shown in other tumor models to correlate with the infiltration of monocytes and M2-macrophages as well [92, 93]. Some investigations have sought to enhance NK migration toward the tumor by genetically modifying NK cells to overexpress chemokine receptors [54, 59, 94]. In one study, Ng et al*.* showed that CAR-NK cells genetically modified to express the chemokine receptor CXCR1 had enhanced migration in vitro and in vivo as compared to control CAR-NK cells [54]. With the subcutaneous hypopharyngeal tumor xenograft model, it was shown that CXCR1-expressing CAR-NK cells had enhanced tumor infiltration and tumor control as compared to control CAR-NK cells [54]. Targeting immunosuppressive components in the TME to re-invigorate NK functions is also under investigation. One major immunosuppressive factor in the TME is the metabolite adenosine, whose production is catalyzed in a sequential manner by the ectoenzymes CD39 and CD73 [43]. Adenosine impairs the anti-tumor functions of both T and NK cells [43]. Wang et al*.* demonstrated that antibody-mediated blockade of CD73 significantly enhanced the anti-tumor activities of NKG2D-enginneered CAR-NK92 in vitro and in vivo, with improved tumor infiltration by CAR-NK cells in vivo [43].

### NK expansion for clinical use

Source and clinical-scale expansion of NK cells with preserved cytotoxic activity are the major challenges for developing clinical-scale NK cell-based therapy. Currently, strategies vary depending on the clinical setting and source of cells. Freshly isolated, activated, or in vitro expanded NK cell populations display phenotypic and functional differences. The differences also arise from activation approaches, such as the choice of interleukins, their combinations, type of feeder cells, and some other factors [95–97]. The current source and characteristics of NK cells for immunotherapies, expansion, and activation approaches are given in Tables 3 and 4.

**Table 3:** Comparison of commonly used allogeneic NK cell sources

NK source	Advantages	Limitations
PB-NK cells	Relatively easy to collect Good in vivo expansion Good clinical track record	Heterogeneous cell population Challenging to genetically modify Can only give one dose
UB-NK cells	NK Progenitors and CD34 + present Higher percentage of *NK cells* The ability to cryopreserve UCB	Heterogeneous cell population
NK92 cells	Defined, homogeneous cell population Easy to expand Easy to genetically modify Can give multiple doses	Tumor cells Irradiated Lack certain receptors, e.g., CD16 limited in vivo expansion
iPSC-NK cells	Defined, homogeneous cell population Circumvent issues with donor sourced cells (donor selection, contaminating T, B) Potential for in depth preclinical testing Defined genetic makeup Easy to genetically modify at iPSC stage Can give multiple doses Can engineer multiple enhancements Don’t need to irradiate- good in vivo survival Suitable for “off-the-shelf” multicancer NK cell therapy	More complicated to produce

**Table 4:** Summary of NK expansion and activation strategies

Stimulation substance	Expansion criteria	Used clinically	Reference	Considerations
**Cytokines alone**: applied separately or in combinations of two IL-2, IL-15, IL-2/IL-15, IL-2/IL-21	~ 5 (two weeks)	Yes	[7, 100, 120]	Generate highly activated NK cells Possibility of dependence on cytokine Expansion is facilitated in the presence of autologous PBMC
IL-2/IL-15/IL-21	~ 8 (two weeks)	No	[119, 121]	Lower rate of NK cell expansion compared to feeder cell Only IL-2 cytokine is GMP-grade
IL-15/IL-18/IL-27	~ 17 (two weeks)	No	[122]	
IL-2, IL-18	~ 500 (two weeks)	No	[123]	
**Autologous feeder cells** OK432, RN-T cells	~ 600 (three weeks)	Yes	[4]	RN-T cells were established by activation PBMC with OKT-3 and RetroNectin FN-CH296
**Autologous feeder + Activating Abs** Anti-CD335 (NKp46) and anti-CD2	~ 3800 (three weeks)	No	Patent, 2013, EP2824112B [153]	CD2 and CD335 coated nanomatrices with commercially available cell stimulation beads (Miltenyi Biotec Kit)
OKT-3 (Anti CD3), anti-CD 52	1,537 (18 days)	Yes	[146]	PBMCs are typically irradiated 25 Gy or more GMP-grade antibody Anti CD3 is available
OKT-3 (Anti CD3)	~ 1000 (two weeks)	Yes	[142, 143]	
Anti CD16	> 500 (two weeks)	No	[147]	
**Allogeneic feeder cells PBMC** + PHA, Ionomycin	~ 100	No	[139]	Without selection final product may contain up to 40% T cell PBMCs are typically irradiated 25 Gy or more
+ ConA	~ 100	Yes	[138]	
+ anti CD3	~ 300	Yes	[144]	
**Allogeneic feeder cells (tumor)** Wilms tumor cell line (HFWT),	~ 113 (two weeks)	Yes	[134]	Feeder can be genetically modified to enhance activation Feeder cells require irradiation and GMP-grade production Final product needs to be feeder free assured
Jurkat	~ 100 (two weeks)	No	[135]	Risk of bacterial and viral contamination derived from feeder cells
**Transformed feeder cells** Epstein-Barr lymphoblastoid cell line (EBV-LCL),	~ 3000 (two weeks)	Yes	[136]	Feeder cells require irradiation Safety considerations associated with feeder
**Engineered feeder** K562 4-1BB + IL15	~ 1200 (two weeks)	Yes	[38, 125–127]	Increased apoptosis of NK cells noted after extensive expansion
Engineered feeder K562 4-1BB + IL21	~ 30,000 (three weeks)	Yes	[128–130]	Greatest rate of expansion reported so far Lower dose of supportive IL-2 required
**Feeder particles** K562 4-1BB + IL21	~ 250 (two weeks)		[141]	Avoids the safety considerations associated with feeder cells Laborious to produce
Group A-Streptococcus and zoledronate	~ 1,560 (three weeks)	No	[107]	> 90% of NK cells. May not require magnetic cell sorting Components IL2, streptococcus and zoledronate are FDA approved

### Donor-derived NK sources

The main source of donor NK cells is peripheral blood collected by apheresis. Using allogeneic NKs for adoptive transfer without expansion after a short overnight incubation with cytokines was shown to be sufficient to activate NK cells and enhance their cell cytotoxicity against tumor targets [86]. However, the dose of NK cells being used for therapy is limited. As the activation and expansion methods are improving, it is becoming possible to prepare increasingly higher dosages of NK cells for adoptive transfer from a single-donor phlebotomy. In order to avoid critical side effects, such as GVHD caused by alloreactive T cells [98] or a passenger lymphocyte syndrome caused by donor-derived B cells [99], purification is recommended for allogeneic NK cells before the expansion to restrict contaminating total T cells to less than 1–5 × 10^5^/Kg. The purification of NK cells is typically achieved by magnetic depletion of CD3-expressing cells and subsequent enrichment for CD56-expressing cells [100] or by fluorescence-activated cell sorting [101].

The umbilical cord blood (UCB) [102–105] or placenta [106] represents other notable sources of NK cells that have been pursued for clinical applications. Both UCB and placenta-derived cells contain some proportion of NK cell progenitors that have the capacity to differentiate into NK cells during maturation and expansion stages. Typically, a dose of UCB or placenta donor can be expended to an amount sufficient for one adoptive transfer procedure. For instance, 21-day NK culture of placenta-isolated NKs yields an average of 1.2 × 10^9^ NK cells with around 80% viability [106] and 1.59 × 10^10^ NK cells with an average purity of 92.37% from UCB [107].

### iPSC-NK cell source

The donor-derived NK cells have certain limitations mainly because of their variability in functional competence and expansion potential. In the clinical setting, each batch requires validation, which results in additional lag time before a patient receives infusion. Moreover, restricted expansion capacity poses a difficulty for improving the efficacy of NK cells through genetic engineering. CAR-NK is one of the approaches in development to overcome this limitation. The approach of using the modified NK cell line CAR-NK92, which can be easily expanded to large dose, has been in clinical trial. However, NK92 is a transformed cell line that has limitations associated with its tumorigenic nature, and cytogenetic abnormalities, thus requiring irradiation for clinical use, which limits its life-time activity.

A novel source of NK cells has emerged to circumvent many of the challenges associated with NK cell therapy. It is iPSC-differentiated NK cells (iPSC-NK). Conceptually, iPSC-NK can provide a homogenously differentiated NK cell population that can be expanded to clinical scale as an “off-the-shelf” supply, overcoming the limitation of the NK-92 cell line. Several groups have demonstrated in vitro derivation of functional NK cells from human embryonic stem cells (hESCs) and iPSCs [108–110]. Typically, the differentiation was induced in embryoid bodies or by OP9 mouse stroma co-culture [111]. Recently, the differentiation method was also established in adherent monolayer cultures [108]. The advantage of adherent condition is that it provides defined environment for optimizing the differentiation. This method has achieved over 15% of CD34^+^ hematopoietic progenitors compared to other methods producing only up to 4% of CD34^+^. The emergence of this technology provided a completely new framework for clinical-scale NK cell production by allowing for the genetic modifications and unlimited expansion to be performed at the pluripotent cell state. CAR-engineered iPSC-NK cells already have demonstrated effectiveness in targeting human tumors in preclinical studies [41]. The other important feature of iPSC-NK cell technology is the ability to manipulate the differentiation strategy, thus shaping the phenotype and functionality of the resulting product. For instance, the enhancement of the *Wnt* signaling pathway with GSK3b inhibitor induces definitive hematopoiesis [112, 113] NK cells developed in such conditions had more pronounced inflammatory cytokine production phenotype, whereas *Wnt* independent NK subsets, similar to primary fetal NK cells, formed a bias for increased cytotoxicity [110]. Such ability to alter the course of differentiation opens a possibility to pursue resident or organ-specific phenotypes of NK cells.

The high proliferation capacity of pluripotent stem cells allows for the introduction of various genetic modifications and for the development libraries of off-the-shelf haplotype-specific cells for treating a range of diseases. There are a number of ongoing clinical trials for cancer immunotherapy using engineered iPSC-NK cells, which are summarized in the following sections.

### Cytokine-induced human NK expansion and activation

Cytokines are the critical components of NK maintenance system and activation as discussed earlier. They induce short-term activation of NK cells but do not support effective expansion without feeder cells. Interleukin (IL)-2 (IL-2) is one of the first and most important cytokines used for NK maintenance and is integral for NK cell survival. It is one of the two cytokines, IL-2 and interferon alpha (IFN-α), approved by the FDA for the treatment of several malignant diseases [114]. IL-2 was used to induce lymphokine-activated killer (LAK) cells, a heterogeneous population of cells consisting primarily of NK, NKT, and T cell for autologous killer cell-based cancer therapy decades ago [115]. The anti-tumor response of LAK cells was shown attributed mainly to NK cells [116]. Noteworthy, IL-2 primarily activates NK cytotoxicity, while supporting proliferation of both NK and T cells. The expansion of NK cells using IL-2 alone is relatively modest and typically results in only several cell divisions in medium containing 1000 U/mL of IL-2 [100]. IL-21 has various effects on human NK cells. On the one hand, it was shown to enhance interferon gamma (IFN-γ) production, cytotoxic functions, and antibody-dependent cellular cytotoxicity (ADCC) responses [117]. On the other hand, it has limited viability support and can trigger proliferative arrest and apoptosis of NK cells at higher dosage (50 ng/mL) [118]. IL-21 has cumulative activation effect in combination with IL-2 [119] or IL-15. It was shown that of CD3-depleted peripheral blood mononuclear cells (PBMC) with IL-21 and IL-15 for 13–20 days resulted in 3.7-fold expansion of NK cells with clinical activity in delaying leukemia progression [120]. The combination of IL-2/IL-15/IL-21 can support up to eightfold expansion of NK cells [121]. Efforts are still ongoing to identify the most optimal cytokine combination for NK cell expansion. Most recent studies showed that ex vivo stimulation of human NK cells with the combination of IL-15/IL-18/IL-27 can achieve 17-fold expansion [122], and that the combination of IL-2 with IL-18 can achieve approximately 500-fold expansion over two-week period [123]. The presence of autologous feeder cells (typically CD3-depleted PBMCs) in culture additionally facilitates NK expansion [124].

It is believed that membrane-bound interleukins are able to stimulate the expansion of NK cells more effectively than the soluble form. A study by Campana and coworkers has shown that stimulation of NK cells with gene-modified K562 expressing the NK-stimulatory molecules 4-1BB ligand and IL-15 induced a median 21.6-fold expansion during a 7-day culture period. It yielded a greater than 1000-fold expansion of NK cells after 3 weeks of culture [38, 125, 126]. An even greater expansion of NK cells, of over 30,000-fold in a period of 3 weeks, was achieved with K562 membrane-bound IL-21 and 4-1BB ligand [127–130]. This protocol created a possibility to generate a substantially higher number of NK cells from a single dose of peripheral blood [131] and is currently in phase I/II clinical trial (NCT01787474) with expanded haploid-identical NK cells for treating relapsed or refractory AML. Moreover, highly cytotoxic NK cells derived using such method are capable of producing endogenous cytokines that improve their survival, proliferation, and function [132].

### Other methods to induce human NK cell expansion

Beyond cytokines, other stimulants, including tumor cells, allogeneic PBMCs, antibodies, and microbiol derivatives, have been explored for enhancing ex vivo NK cell expansion. Exposure of NK to unmodified NK cell-sensitive leukemia cells (K562) stimulates expansion and short-term proliferation [133]. Over 100-fold expansion was achieved with Wilms tumor HFWT [134] and *immortalized* T lymphocyte Jurkat cell lines [135]. Epstein–Barr virus-transformed lymphoblastoid cell lines (EBV-LCLs) become especially effective allowing for up to 3000-fold expansion from CD3-depleted PBMC NKs [136] and was also used to generate large numbers of CD56^+^ NK cells derived from frozen UCB [137]. Chemical stimuli such as *Concanavalin A* (ConA) [138], Phytohemagglutinin (PHA), and ionomycin [139] were also used in combination with irradiated allogeneic PBMCs to facilitate the activation. Although lethal irradiation of feeder cells before use is required, for safety concern, the residual contamination of feeder cells should be assessed. The release criteria should be developed with unique signature to distinguish the feeder cells from expanded NK cells to ensure no feeder cell contamination. For instance, in the use of CD19-modified K562 to propagate NK cells, the contamination was assessed by flow cytometry detection of surface expression of the NK cell endogenous molecule CD32 and the K562 transgene CD19 to distinguish NK cells from the feeder cells [140]. Other methods may include transgene of suicide gene or expression of a fluorescent marker in the feeder cell. Thus, feeder-free approaches are an alternative or a more desirable method to avoid safety concerns associated with the clinical application of cancer cell-derived feeder cells. One approach to address the safety concern is the use of lysed cell product. For example, using the membrane particles of K562 cells with membrane-bound IL-21 and 4-1BB ligand as the feeder has achieved the activation and 250-fold expansion of NK cells after approximately two weeks of ex vivo culture [141].

Stimulating antibodies is typically used along with irradiated allogeneic PBMC to further promote NK activation and expansion. OKT3, an anti-CD3 mAb, has been commonly added to the irradiated autologous PBMC feeder, which can promote the expansion of NK cells up to over 1000-fold [4, 142–144]. The anti-CD3 mAb presumably activates T cells in feeder to secret cytokines which subsequently create a milieu favorable for NK cell expansion [145].

Masuyama et al*.* reported an approximately 1500-fold expansion of NK cells after PBMC stimulation with a combination of anti-CD3 and anti-CD56 mAbs [146]. Using irradiated autologous PBMCs and anti-CD16 mAb, Lee et al*.* demonstrated a more than 500-fold NK expansion with over 98% purity within 2 weeks and a greater than 5000-fold NK expansion over a 3-week period [147].

A more simplified NK cell expansion method has been used by combining group A streptococcus and zoledronate with IL-2 to stimulate UCB-derived mononuclear cells. This method resulted in a 1,560-fold expansion of NK cells with a purity of 92.37% after 21 days of ex vivo culture [107]. This method was advantageous in that it did not require magnetic cell sorting, feeder cells, or multiple cytokines, potentially lowering the cost of production. Furthermore, IL-2, streptococcus A group, and zoledronate have all been approved for human use. Clinical evaluation of safety and efficacy of NK cells under this expansion is warranted.

### Clinical development of NK cell-based cancer therapy

The “off-shelf” NK cell therapeutic product oNKord, the allogeneic partial HLA-matched NK cells derived from UCB-CD34^+^ progenitors, has received an orphan drug designation from EMA and FDA for treating AML patients who were not eligible for allogeneic stem cell transplantation. This approval was based on clinical study demonstrating that oNKord improves survival in year 1 of 80% vs. 35% in the control arm. Recently, the FDA has approved the investigational new drug (IND) for the use of placenta-expanded NK cells (CYNK-001) against glioblastoma (GBM). The success has encouraged many ongoing clinical investigations of NK cell-based cancer therapy alone or in combination with other regimes. Clinicaltrials.gov currently lists over 100 clinical trials of NK cell-based cancer immunotherapy. Herein we highlight current evaluations for hematological malignancies and solid tumors.

### NK cell-based clinical trial for hematological malignancies

The safety and efficacy of allogeneic or autologous donor-derived NK cell-based therapy for treating hematological malignancies, such as AML, have been well established [5, 29, 148]. With the ongoing effort to improve the treatment response and new methods of generating more feasible clinical scales of NK cells, emerging clinical trials are being designed to evaluate these new modalities and to expand their indications. A first-in-human clinical trial of CAR NK-92 cells in 3 patients with relapsed or refractory AML showed that CAR NK-92 can be infused at doses up to 5 billion cells per patient without causing significant adverse effects [149]. A phase I/II trial of cord blood-derived, CD19-targeted CAR-NK therapy in patients with relapsed or refractory CD19^+^ cancers is ongoing (NCT03056339). The interim results showed that 8 out of 11 patients had an objective response to treatment without development of major toxic effects [150]. Table 5 summarizes the clinical trials of NK cell-based therapy for hematological malignancies to date.

**Table 5:** Completed and ongoing clinical trial of NK cell-based therapy for hematological malignancies

Cancer type	Therapy	Phase	NCT	Status
Acute myeloid leukemia	Allogeneic, IL-2 UCB-NK cells	I/II	NCT04347616	Ongoing
	Allogeneic NK cell	I	NCT04220684	Ongoing
	Alloreactive NK cell	N/A	NCT03955848	Ongoing
Poor prognosis non-AML hematologic malignancies	Allogeneic haploidentical NK cell infusions	I	NCT00697671	Completed No outcome reported
Refractory non-B lineage hematologic malignancies	Allogeneic haploidentical NK Cell Infusions	I	NCT00640796	Completed No outcome reported
CD33-positive acute myeloid leukemia	Anti-CD33 CAR-NK cells	I/II	NCT02944162	PMID: 28054442 Found to be effective and prevented both tumor relapses and graft versus host disease
Acute myeloid leukemia & advanced hematological malignancies	Alloreactive, IL-2 activated NK cells	I/II	NCT01220544	Unknown No outcome reported
Hematological malignancy patients who received fate therapeutics	Genetically modified NK cell	N/A	NCT04093622	Ongoing
High-risk tumor and lymphoma	Allogeneic haploidentical NK cell infusion combined with autologous stem cell transplantation	I	NCT02130869	Completed No outcome reported
Pediatric acute leukemia	Activated and expanded NK cells (NKAEs)	II	NCT02074657	PMID: 29477379 Found to be safe and feasible
Chronic Lymphocytic Leukemia (CLL)	NK cell with rituximab and Rhu-GMCSF	I	NCT00383994	Completed No outcome reported
	NK cell	I	NCT02280525	Ongoing
CD19 + Leukemia	Allogeneic anti-CD19 CAR-NK cells	I/II	NCT02892695	PMID: 28054442 Found major improvements in treating leukemia
Multiple myeloma	KIR-ligand mismatched NK cells from a haploidentical donor	I	NCT00089453	Completed No outcome reported
Acute myeloid leukemia & acute lymphoblastic leukemia	Expanded haploidentical NK cells	I	NCT04327037	Ongoing
Ph + acute lymphoblastic leukemia	Autologous NK cell	I	NCT02185781	Unknown No outcome reported
Lymphoma, myeloma, and leukemia	HLA-I haplotype mismatched NK cell	I	NCT00660166	Completed No outcome reported
Chronic myeloid leukemia	NK cell	I/II	NCT03348033	Enrolling
B cell non-Hodgkin’s lymphoma	Cord blood-derived expanded allogeneic NK cells combined with rituximab, high-dose chemotherapy, and stem cell transplant	II	NCT03019640	Ongoing
	CAR.CD19-CD28-zeta-2A-iCasp9-IL15-transduced cord blood NK cells combined with high-dose chemotherapy and stem cell transplant	I/II	NCT03579927	Withdrawn
B cell lymphoma	NK cells with rituximab	I/II	NCT02843061	Completed No outcome reported

^*^ Culture medium of every expansion protocol contains IL-2 or more cytokines

### NK cell-based clinical trial for solid tumors

There are great numbers of clinical trials on NK cell immunotherapy to treat solid tumors. For example, there is an ongoing phase 2 clinical trial to evaluate the safety and efficacy of human HLA-haploidentical hematopoietic cell transplantation (HCT) followed by an early, post-transplant infusion of donor NK cells (NCT02100891). The subjects of the study were patients with high-risk solid tumors, including Ewing Sarcoma, Neuroblastoma, Rhabdomyosarcoma, Osteosarcoma, and CNS tumors. There are also many ongoing clinical trials to evaluate the safety and efficacy of tumor-targeting CAR-NK cells, including using HER2-specific CAR-NK cell to treat advanced or metastatic HER2-expressing solid tumors (NCT04319757), ROBO1-specific CAR-NK cell to treat a broad spectrum of solid tumors (NCT03940820), and MUC1-specific CAR-pNK to treat patients with MUC1-positive relapsed or refractory solid tumor (NCT02839954). MUC1-specific CAR-pNK therapy presented good safety profile and preliminary efficacy in preventing both tumor relapses and graft versus host disease [151]. There are numerous ongoing clinical trials exploring the safety and efficacy of NK-based therapy in combination with other modalities. Phase I/II clinical trials are ongoing to evaluate the safety and efficacy of NK cell combined with nimotuzumab to treat late-stage malignancies (NCT03554889) and of NK cell infusion on patients with advanced malignant tumors following multi-line therapies (NCT03619954). Table 6 summarizes updated clinical trials of NK cell-based therapy for all solid tumors.

**Table 6:** Completed and ongoing clinical trial of NK cell-based therapy for solid tumors

Cancer type	Therapy	Phase	NCT	Status
Gastric cancer	Allogeneic UCB-NK cells	N/A	NCT04385641	Ongoing
	Autologous NK cells combined with Trastuzumab	I/II	NCT02030561	Unknown No outcome reported
Pancreatic cancer	ROBO1 CAR-NK cells	I/II	NCT03941457	Ongoing
	ROBO1 specific BiCAR-NK/T cells	I/II	NCT03931720	Ongoing
Tongue cancer	Cryosurgery combined with NK cells	I/II	NCT02849379	Completed No outcome reported
Esophageal cancer	Cryosurgery combined with NK cells	I/II	NCT02843581	Completed No outcome reported
Laryngeal cancer	Cryosurgery combined with NK cells	I/II	NCT02849314	Completed No outcome reported
Pharyngeal cancer	Cryosurgery combined with NK cells	I/II	NCT02849327	Completed No outcome reported
Cancer lack of MHC-I expression	Autologous-induced T cell like NK cells	I/II	NCT03882840	Ongoing
Small cell lung cancer	Autologous NK cells	II	NCT03410368	Unknown No outcome reported
Non-small cell lung cancer	CCCR-modified NK92 cell	I	NCT03656705	Enrolling
	Hsp70-peptide TKD/IL-2 activated, autologous NK cells	II	NCT02118415	Suspended
	Autologous NK cells	I	NCT03662477	Ongoing
	Cryosurgery combined with allogeneic NK cells	I/II	NCT02843815	PMID: 28508945 Showed preliminary efficacy
	Cetuximab combined with NK	I/II	NCT02845856	Completed No outcome reported
Renal cancer	Cryosurgery combined with NK	I/II	NCT02843607	Completed No outcome reported
Breast cancer	Cryosurgery combined with NK	I/II	NCT02844335	Completed No outcome reported
	Trastuzumab combined with NK	I/II	NCT02843126	Completed No outcome reported
Ovarian cancer	Cryosurgery combined with NK	I/II	NCT02849353	Completed No outcome reported
Cervical cancer	Cryosurgery combined with NK	I/II	NCT02849340	Completed No outcome reported
Neuroblastoma	Autologous NK combined with antibody ch14.18 and lenalidomide	I	NCT02573896	Ongoing
	Allogeneic NK combined with the anti-GD2 antibody	I/II	NCT03242603	Unknown No outcome reported
Liver cancer	Cryosurgery combined with NK	I/II	NCT02843802	Completed No outcome reported
	Allogeneic NK	I	NCT01147380	Completed No outcome reported
	Irreversible electroporation and autologous NK	I/II	NCT03008343	Completed No outcome reported
	Cryosurgery combined with NK	I/II	NCT02849015	Completed No outcome reported
High-risk solid tumor	Allogeneic NK cells with human leukocyte antigen (HLA)-haploidentical hematopoietic cell transplantation (HCT)	II	NCT02100891	Ongoing
Metastatic HER2-expressing solid tumor	Allogeneic ACE1702 (anti-HER2 oNK cells)	I	NCT04319757	Ongoing
Solid tumor expressing ROBO1	ROBO1 CAR-NK cells	I/II	NCT03940820	Ongoing
MUC1-positive solid tumor	Allogeneic anti-MUC1 CAR-pNK cells	I/II	NCT02839954	PMID: 28054442 Show preliminary efficacy
Late-stage malignancies	Autologous NK cell combined with nimotuzumab	I	NCT03554889	Unknown No outcome reported
Recurrent malignant solid tumor	NK cell combined with Bevacizumab	I/II	NCT02857920	Completed No outcome reported
	NK cells	I	NCT03619954	Unknown No outcome reported
Pediatric solid tumor	Autologous NK cell	I	NCT01875601	Completed No outcome reported

## Conclusions

NK cell represents a specialized immune effector cell population equipped with fast-acting and potent anti-tumor capacity. The concept of adoptive NK cell cancer immunotherapy was proven a decade ago from pioneering clinical studies against hematological malignancies. The difficulty to obtain large quantity of NK cells, to expand to clinical scale ex vivo, and to sustain in vivo survival and activity of infused NK cells has encumbered the progress. With the new tools of iPSC-NK and genetic engineering approach as well as new understandings of NK cell biology, it is the time to re-explore the therapeutic potentials of NK cells. Although challenges exist, perspectives are enticed by currently approved NK cell-based therapies and emerging pre-clinical and clinical studies.

## Data Availability

Not applicable.

## Abbreviations

NK: Natural killer
MHC I: Major histocompatibility complex I
CAR: Chimeric antigen receptor
IL: Interleukin
ADCC: Antibody-dependent cell cytotoxicity
AML: Acute myeloid leukemia
iPSC: Induced pluripotent stem cell
LAK: Lymphokine-activated killer
